# Assessment of the potential cerebellar toxicity of gold nanoparticles on the structure and function of adult male albino rats

**DOI:** 10.1042/BSR20222255

**Published:** 2023-08-31

**Authors:** Shimaa Mohammad Yousof, Horeya Erfan, Shaimaa A. Shehata, Marwa M. Hosny, Karima El-Sayed

**Affiliations:** 1 Department of Medical Physiology, Faculty of Medicine in Rabigh, King Abdulaziz University, Jeddah 21589, Saudi Arabia; 2 Department of Medical Physiology, Faculty of Medicine, Suez Canal University, Ismailia 41522, Egypt; 3 Department of Histology and Cell Biology, Faculty of Medicine, Suez Canal University, Ismailia 41522, Egypt; 4 Department of Forensic Medicine and Clinical Toxicology, Faculty of Medicine, Suez Canal University, Ismailia 41522, Egypt; 5 Department of Medical Biochemistry and Molecular Biology, Faculty of Medicine, Suez Canal University, Ismailia, Egypt; 6 Oncology Diagnostic Unit Lab, Faculty of Medicine, Suez Canal University, Ismailia, Egypt

**Keywords:** Au NPs, Cerebellar toxicity, narrow beam test, OFT, Rgc32, Trkb

## Abstract

Background: The regular use of gold nanoparticles (Au-NPs) may increase the likelihood of human exposure to these nanoparticles (NPs) and raises concerns about toxicity. Aim: This study investigated the short-term impact of exposure to Au-NPs on inducing cerebellar pathology in rats, and whether the dose or duration of exposure was more important. Methodology: The study used two concentrations of Au-NPs (25 and 50 particles per million) and 18 rats were randomly assigned to three groups. Assessments of the animals were done via behavioral, gene expression, histological, and immunohistochemistry analyses. Results: Both concentrations of Au-NPs caused cerebellar pathology, as assessed through the investigation test battery. The Au-NPs_50_ group displayed more injury and decreased mobility compared with the control and the Au-NPs_25_ group. The Au-NPs_25_ group showed an increase in supported rearing and significant up-regulation of the Rgc32 gene compared with the control. The Trkb gene was insignificantly up-regulated in both Au-NPs groups compared with the control. Conclusion: The study indicates that exposure to Au-NPs can cause cerebellar pathology in rats and that the toxicity is more dependent on dose than the duration of exposure. These findings have significant implications for the safe use of Au-NPs in various applications.

## Introduction

Nanotechnology is defined as the study and application of structures ranging in size from 1 to 100 nm. Nanotechnology is a combination of chemical engineering, mechanical engineering, microelectronics, electrical engineering, material sciences, and biological screening. Nanotechnologies are the creation, planning, categorization, and application of devices, structures, and systems at the nanoscale scale. There are now over 300 declared nanotechnology goods on the market [1]. Nanomedicine has made great advances in the medical diagnosis, prevention, and treatment of illnesses [2]. Progress in this subject has enabled the development of nanoparticles (NPs) capable of delivering medications to particular regions and improving the pharmacokinetic profile of several bioactive substances with biological uses [3]. Gold nanoparticles (Au-NPs) have special properties, such as ease of synthesis, chemical stability, and specific optical properties [4]. Modern Au-NPs’ biomedical applications include drugs and gene delivery, bioimaging, immunotherapy biocarriers, radiotherapy, photothermal, and nanocosmetics products [5–7]. Recently, numerous research reports have documented the promising role of Au-NPs in cancer diagnosis and therapy [8,9]. Furthermore, they are being used in the drink and food industries [10]. Other valuable environmental applications involve solar cells liquid, water cleansing, and pollution management [11]. The widespread use of Au-NPs regularly may enhance the likelihood of human exposure to these NPs and raises concerns about the possibility of toxicity when the NPs are redistributed and stored in certain vital organs [12–14]. Despite the significant contribution of Au-NPs to medical and commercial advancement, there is scarce information on short- and long-term health effects on different human tissues, implying the need for a thorough investigation [13,15]. The toxicological profile of Au-NPs is defined by their chemical structure, shape, size, aggregation, and surface coating [16]. Although Au-NPs have low toxicity in comparison with other NPs, some animal research revealed the toxicological effects of Au-NPs on the liver [17], kidney and spleen [18], fibroblasts [19], brain [20], and DNA fragmentation [21]. An earlier study reported a noticeable difference in distribution between the 10 nm particles and larger particles. The 10 nm particles were present in numerous organs, including the blood, liver, spleen, kidney, testis, thymus, heart, lung, and brain. Conversely, the larger particles were only detected in the blood, liver, and spleen. These results suggest that the tissue distribution of Au-NPs is size-dependent, with the smallest 10 nm particles exhibiting the most widespread organ distribution [22]. Another study analyzed the effect of Au-NPs size on their accumulation in the brain tissue. The results of the pooled and stratified analysis showed that the size of the Au-NPs affected their accumulation in the brain tissue, with 10 nm particles showing a higher accumulation compared with larger sizes. Some larger Au-NPs (up to 120 nm) were also able to cross the blood–brain barrier (BBB) after producing a temporary increase in BBB permeability. However, Au-NPs size not only governs their passage through the BBB but also affects their uptake by cells. Some studies have found that 50 nm Au-NPs are the best size for cell uptake, while another study found that 20 nm and smaller Au-NPs were better [23].

Au-NPs have been shown to accumulate with significant concentrations in the liver and spleen, causing further damage to the organism and can cross the BBB, causing accumulation in many areas of mice brain [24]. A systematic review and meta-analysis study investigated the distribution of Au-NPs in the brain over time after injection. The results showed that the peak amount of Au-NPs in the brain occurred one hour after injection and gradually decreased over time, with detectable Au-NPs still present up to 4 weeks after injection. Stratified analysis confirmed the pooled meta-analysis results, with a significant decrease in Au-NPs in the brain after 72 h compared with before 12 h [23]. However, it is important to note that the study’s findings are limited to the specific parameters of the study and further research is needed to fully understand the behavior and potential effects of NPs in the brain. A study found that gold concentrations in all brain regions and cerebrospinal fluid showed a similar time-dependent profile after the injection of Au-NPs. The concentration of gold increased rapidly during the first 10 h and then decreased to low levels 24–48 hours after injection. These changes in gold concentration were due to variations in the concentration of gold in the blood, which is the source of Au-NPs in all organs. Based on these results, the study estimated the biological half-life of Au-NPs in rats to be 12.9 ± 4.9 h [25].

The brain is vulnerable to oxidative stress due to its high oxygen utilization, high levels of polyunsaturated fatty acids, and abundance of redox-active transition metal ions [26]. Toxicity studies have found that Au-NPs induced neuro-oxidative damage and cell death via the generation of reactive oxidative stress (ROS) and a reduction of antioxidant enzymes [18,20,27]. It has been established that Au-NPs caused DNA damage, which is a cause of genotoxicity [28]. The ability of Au-NPs to stabilize and allow direct access to the surface of Au, either for catalytic activity of the uncovered surface or for direct association with biological molecules, could explain their cytotoxic potential [29]. Previous studies showed that Au-NPs induced cell death through necrosis and apoptosis with Au-NPs of size 1.4 and 1.2 nm [13]. Additionally, inflammation and apoptosis of hepatic tissue were detected in mice after intravenous administration of Au-NPs [30]. Furthermore, Au-NPs cause a significant decrease in neurotransmitter levels such as dopamine and serotonin, implying a possible alteration in the behavior of the treated animals [20].

The use of Au-NPs is widespread and their potential harmful effects on human health are a cause for concern. Therefore, it is essential to investigate the impact of Au-NPs on various organs, including the cerebellum, which has received little attention in previous studies. The objective of this research is to examine the potential adverse effects of short-term oral administration of two different doses of Au-NPs and two different time frames on the cerebellum of adult albino rats. The present study is innovative in that it seeks to determine whether the dose or the duration of exposure is more significant in inducing cerebellar pathology in the short term. Unlike previous studies that have investigated either dose–response or duration effects separately, the present study aims to investigate both factors concurrently.

## Methods

### Animals

Healthy adult male albino rats (*n*=18, age: 8 ± 1 weeks, 220–225 g) were purchased (Source: The Ophthalmic Research Institute in Giza, Egypt). This experimental observational study was conducted in the animal house of the Faculty of Medicine, Ismailia, Egypt.

**Figure 1:**
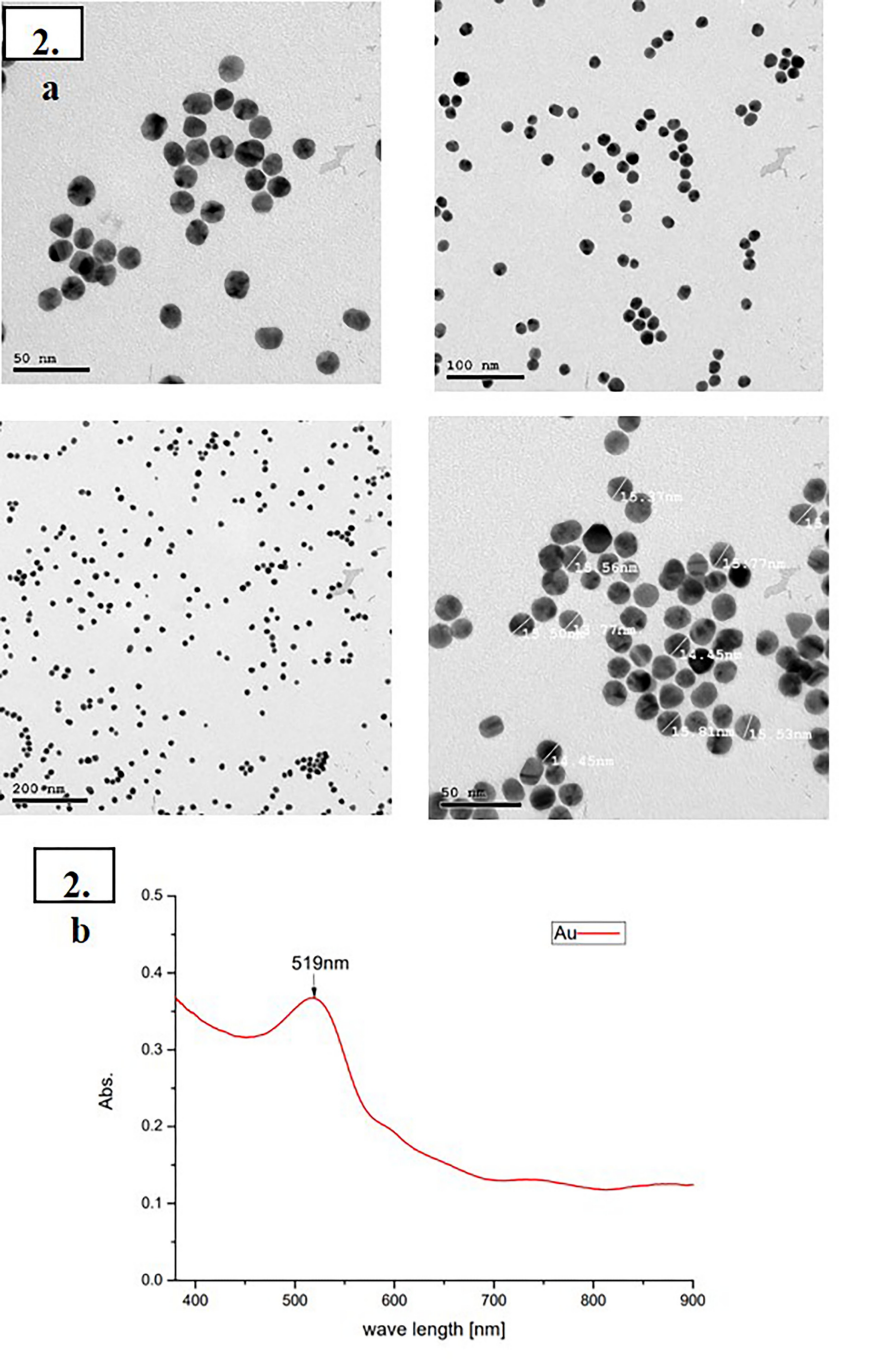
Characterization of AuNPs (**A**) TEM characterization, revealing Au-NPs spherical in shape and the range. (**B**) UV-Vis reveals homogeneity in the size distribution of Au-NPs.

One week before the start of the experiment, rats were kept to adapt in the animal house. The housing of the animal was in a controlled environment (temperature: 25 ± 2°C, humidity: 55 ± 5%, natural light, and dark cycles). Standard chewing meals and room-temperature water were freely available to the animals, Figure 1. Experiments were performed with the permission of the Research Ethics Committee of the Faculty of Medicine, Suez Canal University. All experiments will be conducted by the principles of laboratory animal care (No: 4865#).

### Materials

#### Au-NPs preparation

The nanogold preparation products were purchased (St. Louis, MO, U.S.A.), and the NPs were prepared by an expert professor at the Center of Excellence, College of Medicine, Suez Canal University. One commonly used method for synthesizing Au-NPs involves the reduction of gold chloride using trisodium citrate. In this method, trisodium citrate acts as both a reducing agent and a stabilizer. Initially, citrate ions form a layer over the Au-NP surface, helping to disperse the particles and prevent them from clumping together. The reduction process leads to the formation of Au-NPs, which are stabilized by the citrate ions, preventing them from aggregating. To synthesize the Au-NPs, 25 ml of 1.0 mM of chloroauric acid (HAuCl_4_) was added to a 50 ml beaker on a stirring hot plate. The solution of chloroauric acid (HAuCl_4_) is heated and then an aqueous solution of 2.5 ml of trisodium citrate as a capping agent was added while stirring vigorously. Au-NPs are formed through crystallization, resulting in a stable, well-dispersed solution of Au-NPs [31,32]. The prepared nanogold solution was used in two concentrations by dilution using distilled water (concentrations of 25 and 50 parts per million; ppm) and were kept in a container covered with aluminum foil in a dark place.

#### Characterization of Au-NPs

##### Transmission Electron Microscopy (TEM)

A highly specialized laboratory in Egypt utilized Transmission Electron Microscopy (TEM) to evaluate the radius and morphological properties of Au-NPs. TEM results indicate sphere-shaped Au-NPs particles with sizes ranging from 13.77 to 15.77 nm as shown in Figure 1 (average: 15.22 nm). On a stirring hot plate, 25 ml (0.001 M) of 1.0 mM HAuCl_4_ was added to a 50 ml beaker. After adding a magnetic stir bar, the solution was brought to a boil. The flask containing gold salt was positioned on a hot plate and wrapped with aluminum foil while the solution heated up. With vigorous stirring, 2.5 ml (0.0388 M) of Tri-sodium citrate solution as a reducing agent was added over the boiling gold solution [33].

##### Ultraviolet-Visible (UV-Vis) spectroscopy analysis

**Figure 2:**
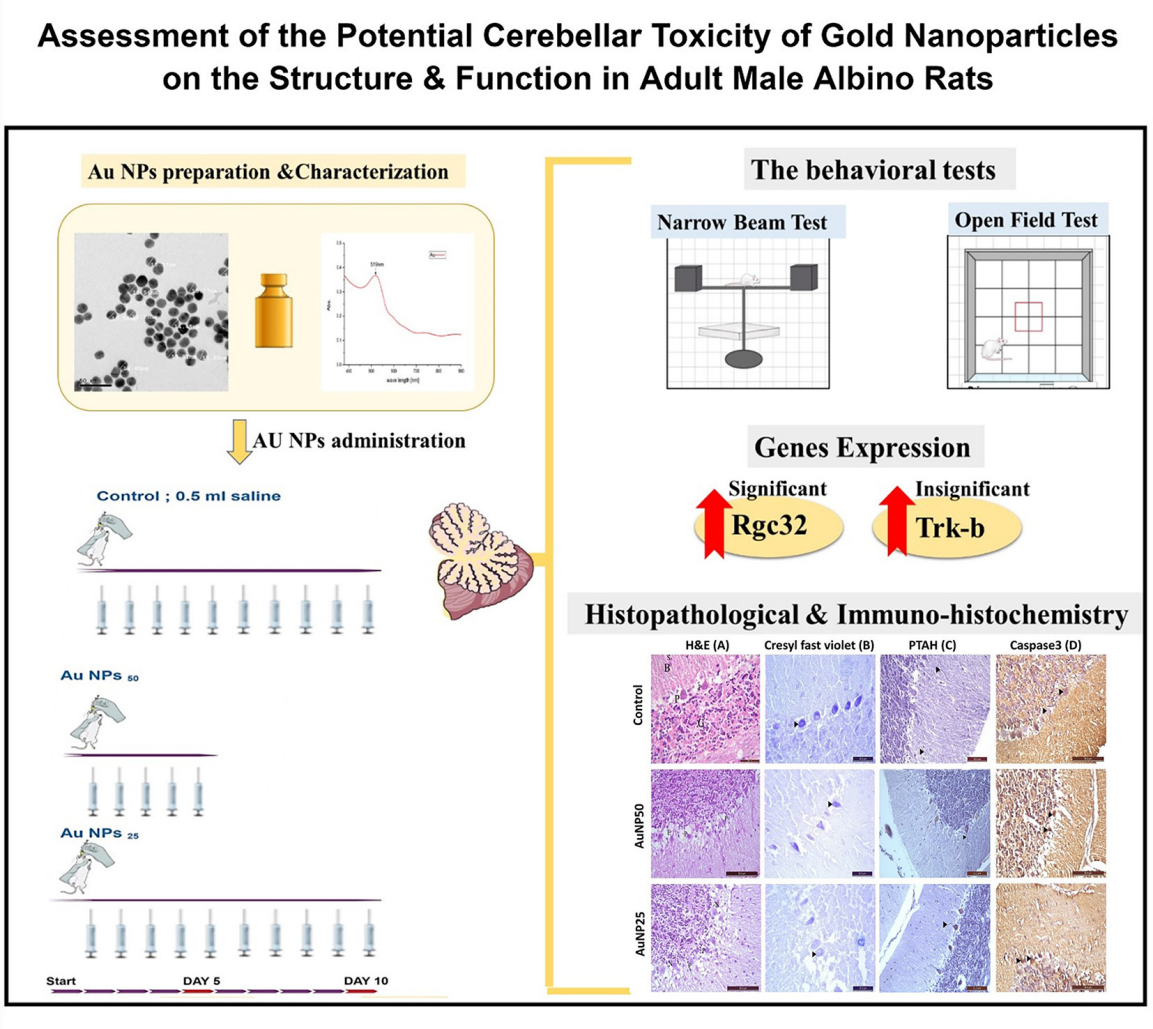
A graph summarizing the steps and investigations of the study The upper left side reveals the nanoparticles characterization. The lower left side reveals the time line of the experiment and the experimental groups. The right part showsg the behavioral tests, gene analysis and histological assessments carried out for the animals.

Ultraviolet-Visible (UV-Vis) analysis was used to determine the diameter and morphological properties of Au-NPs. The wavelength of maximum absorbance was 519 nm. The narrow wavelength distribution demonstrates NP size homogeneity; Figure 2.

### Experiment design

At the start of the experiment, 18 rats were randomly assigned to three groups (*n*=6 each). Control group was given oral dose of 0.5 ml of saline daily; Au-NPs_50_ group was given a single daily dose of NPs solution (0.5 ml of a solution containing 50 ppm for 5 days Au-NPs) [34–36]. Au-NPs_25_ group was treated with a single daily dose of Au-NPs solution (0.5 ml of a solution containing 25 ppm Au-NPs for 10 days) [34–36]. The treatments in all groups were via oral gavage at 11 a.m [37]. To avoid causing undue harm to the rodents, oral gavage was formed by a trained professional using metal needles for feeding (Gauge: 16G; length: 3 inches). Before being sacrificed by cervical dislocation, animals were anesthetized with an intraperitoneal injection of thiopental Na (40 mg/kg) [38]. Testing sessions of behavioral tests were done at day 5 followed by sacrifice in Au-NPs_50_ group. The same procedures were repeated at day 10 for NPs_25_ and control groups. Rat cerebella were fixed into formalin-fixed, paraffin-embedded blocks for further investigations.

### Behavioral analysis

#### Narrow (or balance) beam test (NBT)

**Figure 3:**
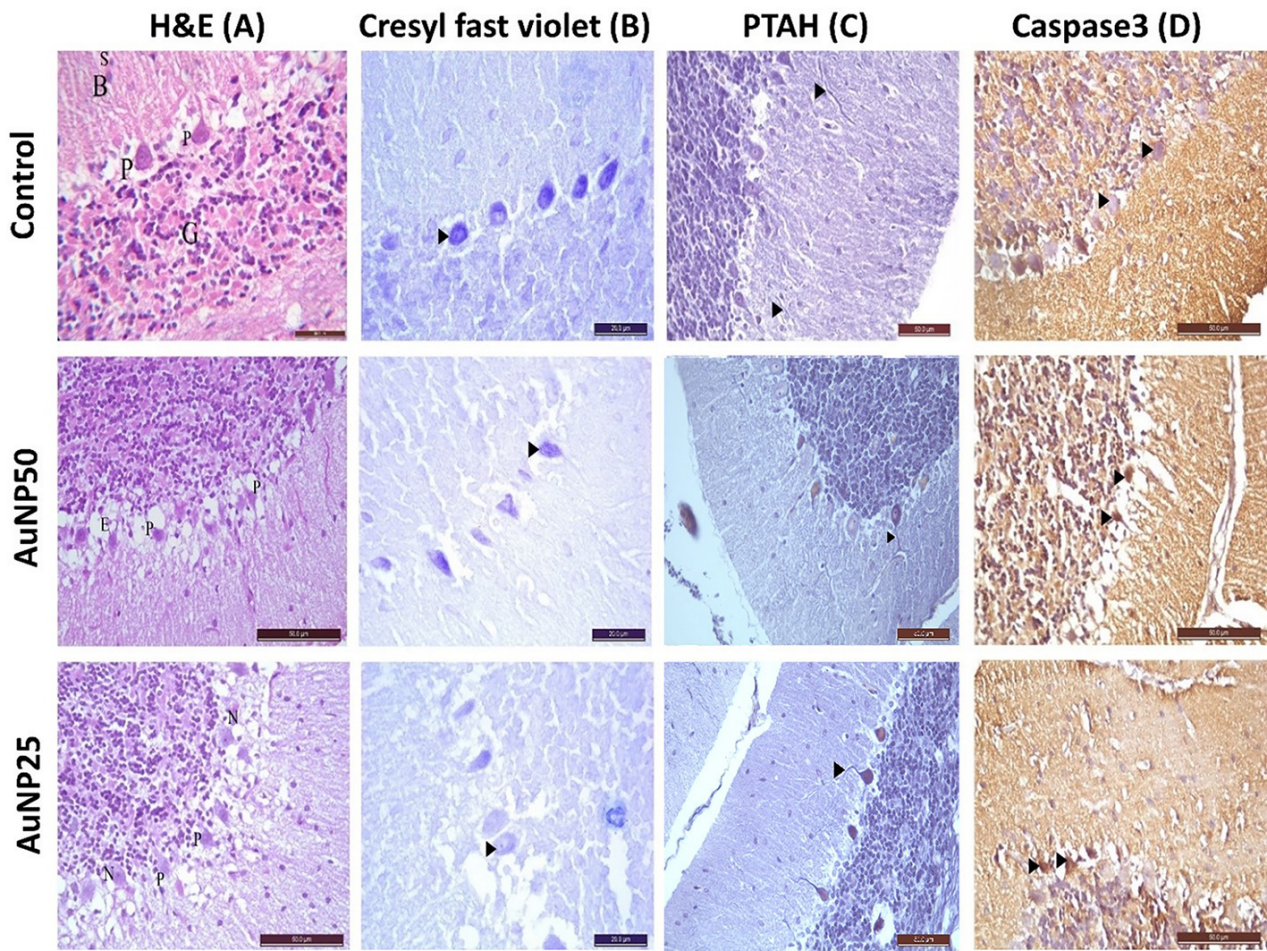
(**A**) A photomicrograph of a section in Au-NPs induced cerebellar cortex of rats from the different groups (H&E: ×400) Au-NP_50_: gold nanoparticles 50 ppm for 5 days, Au-NP_25_: 25 ppm Au-NPs for 10 days: Control group: showing scattered small stellate (S) and basket cells (**B**) in the molecular layer Large pyriform cells with vesicular nuclei (P) are shown in the Purkinje cell layer. Crowded small cells with deeply stained nuclei (G) in the granular layer. Au-NP _50_ group: showing Purkinje cells (P) are widely displaced, shrunken irregular and distorted that leave empty spaces in intercellular spaces (E). Au-NP_25_ group: showing distorted Purkinje cells and shrunken (P) and others are appeared normal (N). (**B**) A photomicrograph of a section in the cerebellum stained with cresyl fast violet ×400 of rats from different groups: Control group (I) showing purple Nissl’s granules in the perikarya of Purkinje cells with deeply stained nuclei (►). Au-NP_50_ group: showing marked decreased purple Purkinje granules in the perikaryal of Purkinje cells (►). Au-NP_25_ group: showing moderate decreased purple Nissl’s granules in the perikarya of Purkinje cells (►). (**C**) A photomicrograph of a section in the cerebellum stained with PTAH ×400 of rats from different groups: Control group (I) showing blue discoloration of Purkinje cells dendrites in the molecular layer (►). Au-NP_50_ group and N25 group showing in of Purkinje cells dendrites (►) are similar to control group. (**D**) A photomicrograph of a section in the cerebellum immunostained with Caspase3 ×400 of rats from different groups: Control group (I) showing negative immunostaining reaction in the nucleus of Purkinje cells (►). Au-NP_50_ group: showing marked immunostaining reaction in the nucleus of Purkinje cells (►). Au-NP_25_ group:showing moderate immunostaining reaction in the nucleus of Purkinje cells (►) and presence of others negative immunoreaction of Purkinje cells.

***Set:*** NBT was used to assess the coordination and balance of the animals. The set is composed of two cubical wooden chambers (height: 28 cm, width: 18 cm, depth: 18 cm) connected with a straight beam (length: 105 cm, width: 4 cm, height: 4 cm). One of the chambers was black to attract the animal to get into it. The parts of the equipment were supported and elevated approximately 80 cm from the ground. For animal protection from hurt during fall, a sponge pad was placed below the beam [39,40]; Figure 3.

***Experiment:*** The animal must remain upright and walk across an elevated narrow beam to a safe platform in this test. This test is conducted over 3 days: 2 days of training and 1 day of testing. The task had a 2-min time limit. The animal should traverse the beam from one side to the other [39,40]. On test day, the times taken to cross each beam are recorded. The average of two successful trials in which the mouse did not slow down or stop on the beam. The trials were video tapped for further analysis.

***Analysis:*** The time the animal takes to traverse the beam and the number of paw slips that occur during the process are used to quantify performance on the beam. A neurological scoring system which scores the failure to cross the beam as a ‘1’ and the ability to traverse the beam normally with both paws on the beam surface and less than two-foot slips as a ‘7,’ is another way to assess beam walking. A slip occurs when a foot falls off the top of the beam. A score of ‘3’ in this indexing system corresponds to dragging limbs across the beam. An animal that receives a score of 1 in two trials is not retested [40].

#### Open Field Test (OFT)

***Aim:*** It was used to assess locomotor behavior of the animal.

***Set:*** A non-porous white cubic arena (length: 100 cm, width: 100 cm, height: 30 cm) was used. The test protocol followed Seibenhener and Wooten’s (2015) guidelines [41].

***Experiment:*** An hour before the test, the animals were brought to the test room to habituate. To avoid the animals from escaping to the arena’s unlit area, the room was lit with an even, upright illumination. The animals were placed in the center of the arena and given 5 min to freely roam around. A digital camera was used to record rat motion and behavior in the arena for later data analysis. After each subject testing trial, the arena was cleaned [41]; Figure 3.

***Analysis:*** Grooming, rearing behavior, central preference and time of immobility were the parameters used to blindly assess animal performance in open field test (OFT) [42].

### Histopathological and immunohistochemical assessment

Cerebellum was obtained from each animal. The specimens were fixed in 10% neutral buffered formalin for 24 h at room temperature (23–28°C) and were processed to prepare 5 µm thick paraffin sections. The obtained sections were stained using hematoxylin & eosin stain (H&E) for the general architecture of the cerebellum [43]: Cresyl fast violet for Nissl’s granules of the Purkinje cells, Mallory’s phosphotungestic acid hematoxylin (PTAH) staining for dendritic length of the Purkinje cells [44], and immunohistochemical assessment (anti- caspase-3) for apoptosis (Catalog No. A11953, http://abclonal.com). Qualitative and quantitative assessments were done for histological changes in the cerebellum.

In the cerebellum, the examination of five high power fields (×400) in 10 serial sections from each animal of all studied groups was used for qualitative assessment. All images were captured with an Olympus® CX21microscope and a calibrated standard digital microscope camera.

The severity of tissue damage (scoring) is as follows. The severity of changes in the cerebellum was quantified from none (-) to severe (+++) based on Purkinje cells, which can be widely displaced, shrunken irregularly, and distorted, leaving empty spaces in intercellular spaces. (-), no damage; (+), minimal displacement, shrunken irregular, and distortion (<5%); (++), moderate displacement, shrunken irregular, and distortion (5–20%); (+++), widespread displacement, shrunken irregular, and distortion (>20%). The computer software ImageJ was used to perform quantitative assessment. The optical density of Nissl’s granules and anti-caspase3 immunostaining activity were both measured.

### Relative Expression of *Rgc32* and *Trkb* Genes

Total RNA was extracted from the formalin-fixed, paraffin-embedded cerebellum tissue using The RNeasy FFPE Kit (Qiagen, Hilden, Germany, cat. no. 73504) and following the manufacturer’s protocol. The NanoDrop ND-1000 spectrophotometer (Wilmington, DE, U.S.A.) was used to determine the concentration and purity of RNA and cDNA. ABT H-Minus c-DNA synthesis kit (Biotechnology, Egypt) and a thermocycler (BIOMETRA®, LA, U.S.A.) were used for reverse transcription. The gene expression of the for Rattus norvegicus regulator of cell cycle (*Rgc32*) and neurotrophic receptor tyrosine kinase 2 (Ntrk2) or (*Trk-b*) genes were determined using an Applied Biosystems Step One TM Real-Time PCR apparatus with the PCR conditions; Table 1. These Primer’s sequences (willowfort, U.K.) were engineered and examined by utilizing web Bioinformatics tools; Table 2. The 25 μl of PCR included 7.5 μl of RT product (diluted to reach a concentration of 20 ng cDNA), 1 μl of RNase-free water, 12.5 μl of SYBR green Maxima SYBR Green qPCR master mix (Thermofisher Scientific, U.S.A., cat. No. K0251), Maxima SYBR Green qPCR master mix (Thermofisher Scientific, U.S.A., cat. No. K0251), Maxima SYBR Green qPCR master mix (Thermofisher Scientific, U.S.A., cat. No. K0251), Maxima SYBR Green qPCR master mix (Thermofisher Scientific, U.S.A., cat. No. K0251), 2 μl of diluted 1:9 (concentration 10 pg) forward primer (willowfort, U.K.) and 2 μl of diluted 1:9 (concentration 10 pg) reverse primer (willowfort, U.K.). Every target gene was normalized (endogenous β-actin). The 2^−ΔΔCT^ Method was utilized for calculating the relative expression of genes [45].

**Table 1:** Thermal cycling protocol for StepOne™ Real-Time PCR System

Step	Temperature	Time	Number of cycles
*Initial denaturation*	95°C	10 min	1
*Denaturation*	95°C	15 s	40
*Annealing*	56°C	30 s	
*Extension*	72°C	30 s	

Note: A melting curve analysis was then performed to rule out primer dimer.

**Table 2:** Shows *Rattus norvegicus* primer nucleotide sequence used in real-time PCR

Gene symbol	Forward primer Sequence (5′->3′)	Reverse primer Sequence (5′->3′)
***Rgcc (Rgc32)***	ACTCCTCGGAAAGCCAAATTA	GAATCTCAAACTCCTTGCTTCAC
***Trk-b (Ntrk2)***	GCCTGTGTATGAGAAGGGAAAG	TCACTCCTGCTGTGCTTTATG
***β-Actin***	TGTGACGTTGACATCCGTAAAG	GGCAGTAATCTCCTTCTGCATC

Rgc32 (Rggc), regulator of cell cycle; Trk-b (Ntrk2), neurotrophic receptor tyrosine kinase 2.

### Data analysis

The data were examined using IBM SPSS statistical software version 23 [46]. The results were expressed as the mean ±standard deviation, and the means were compared using ANOVA and the post-hoc LSD test.

## Results

### Behavioral tests analysis

#### Narrow Beam test (NBT)

The ANOVA analysis of the NBT scores, as presented in Table 3, did not reveal any statistically significant differences among the groups (*P*>0.05), indicating a lack of discernible variations in locomotion between them. Nonetheless, it should be noted that certain rats exhibited freezing behavior during the test, which could be indicative of the presence of anxiety.

**Table 3:** Assessment of behavioral functions in rat after Au-NPs administration

Group parameters		Control		Au-NPs _50_		Au-NPs _25_		*P-*value#
		Mean	SD	Mean	SD	Mean	SD	
**NBT**	**Foot slips (No)**	0.00	0.000	0.00	0.000	0.00	0.000	–
	**Time to cross (s)**	6.33	2.944	6.67	1.366	4.50	2.036	0.139
	**Score**	1.00	0.000	1.00	0.000	1.00	0.000	
**OFT**	**Immobility (s)**	124.17	85.815	244.00	62.161	174.33	35.674	0.019*
	**Central preference (s)**	2.83	3.601	4.67	5.955	10.17	6.398	0.114
	**Supported rearing (No)**	6.00	2.828	1.67	1.366	8.17	5.382	0.021*
	**Grooming (No)**	3.50	2.429	1.33	.816	2.83	1.602	0.122

Au-NP_50,_ gold nanoparticles 50 ppm for 5 days; Au-NPs_25_, 25 ppm Au-NPs for 10 days; NBT, narrow (balance) beam test; OFT, open field test; #, ANOVA test; *P<0.05.

#### Open field test

The ANOVA test revealed a significant difference among groups with respect to immobility and unsupported rearing in the OFT (*P*: 0.019 and 0.021, respectively). Post-hoc LSD analysis showed that there was a statistically significant difference between the control group and the Au-NPs_50_ group in terms of immobility (mean ± SD: 124.17 ± 85.815, 244 ± 62.161, respectively, *P*<0.01), but no significant differences were found between the Au-NPs_25_ and Au-NPs_50_ groups (*P*>0.05). As for supported rearing, the post-hoc LSD analysis revealed a significant difference between the control group and the Au-NPs_50_ group (mean ± SD: 6.0 ± 2.828, 1.67 ± 1.366, respectively, *P*=0.05). Moreover, there was a statistically significant difference between the Au-NPs_25_ and Au-NPs_50_ groups in terms of supported rearing (mean ± SD: 1.67 ± 1.366, 8.17 ± 5.382, respectively, *P*<0.01). However, there were no significant differences observed in other parameters (*P*>0.05), as presented in Table 3. The results indicate that the high dose had a detrimental effect on rearing behavior, as evidenced by a decrease compared with the control group. Furthermore, the low dose group demonstrated an increase in rearing behavior relative to the high dose group.

### Histopathological and immuno-histochemistry

**Figure 4:**
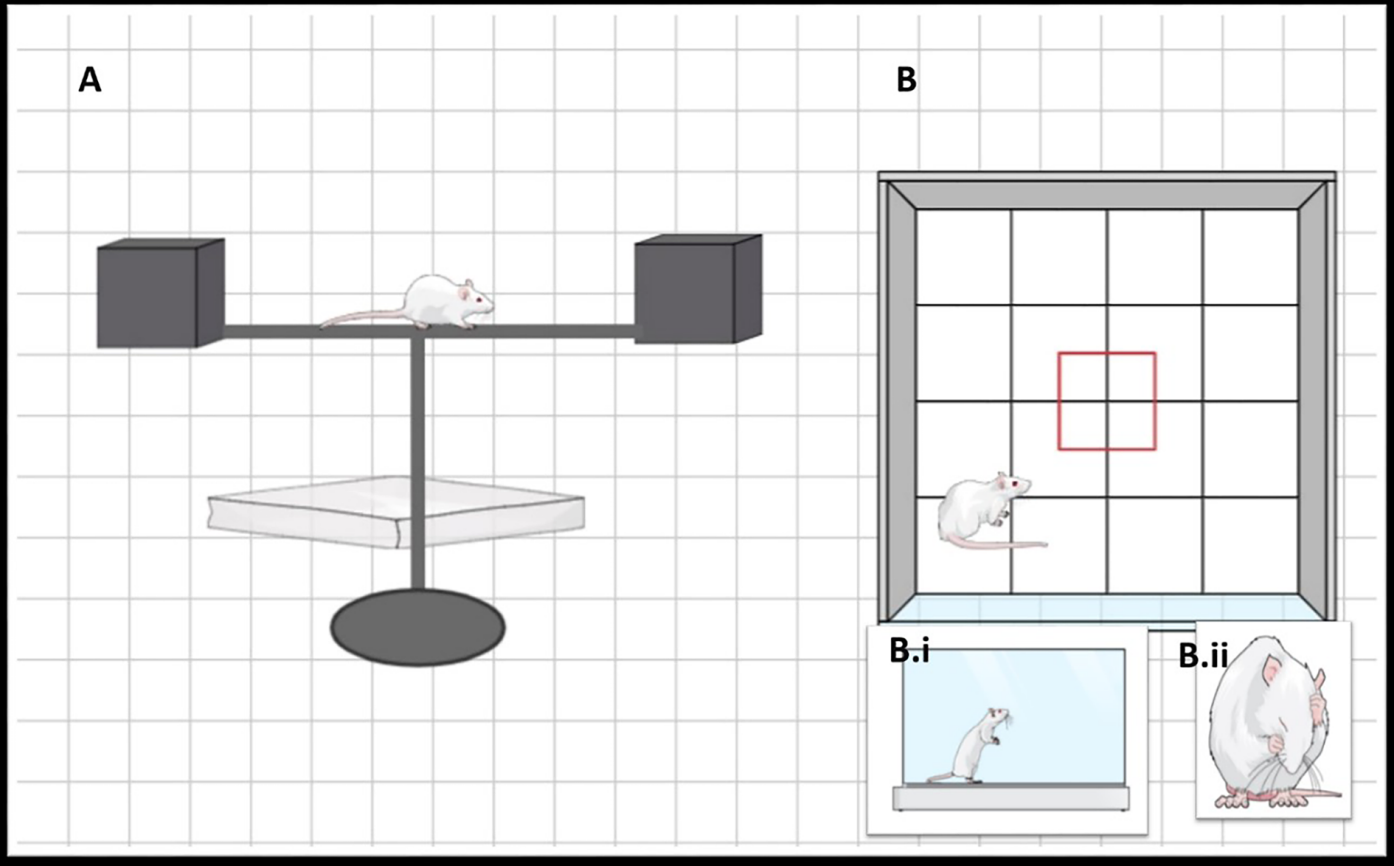
Graphical represenation of the behavioral tests **(A**) Narrow beam test; showing the chambers, the beam and the sponge pad. (**B**) Open field test; showing the arena of the test. (B)i showing the rearing behavior, while (B)ii showing the grooming behavior.

Upon microscopic examination, both groups treated with Au-NPs exhibited histopathological abnormalities in the cerebellum when compared to the control group. Specifically, Purkinje cells were observed to be displaced, shrunken, and distorted, with a corresponding decrease in purple Nissl’s granules in the perikarya of these cells (as detailed in Table 4). Furthermore, a positive caspase 3 reaction was noted in the nucleus of Purkinje cells, with varying degrees of immunostaining reaction (as presented in Table 5). These histopathological changes were found to be more dependent on dose than on time, as demonstrated in Figure 4.

**Table 4:** The mean and standard deviation (SD) of the optical density of Nissl’s granules in the different experimental groups

Group	Mean ± SD
**Control**	10.99 ± 0.7
**Au-NP_50_**	3.349 ± 0.2
**Au-NP_25_**	5.42 ± 0.6

Au-NP_50_, gold nanoparticles 50 ppm for 5 days; Au-NP_25_, 25 ppm Au-NPs for 10 days.

**Table 5:** The mean and standard deviation (SD) of the optical density of caspase 3 immunostaining in the different experimental groups

Group	Mean ± SD
**Control**	13.523 ± 0.6
**Au-NP_50_**	19.179 ± 0.4
**Au-NP** **_25_**	16.772 ± 0.3

Au-NP_50,_ gold nanoparticles 50 ppm for 5 days; Au-NP_25_**_,_** 25 ppm Au-NPs for 10 days.

### Relative Expression of *Rgc32* and *Trk-b* Genes

The expression of the *Rgc32* gene was found to be significantly increased in both Au-NPs groups compared with the control group (*P*=0.05), with a greater increase observed in the Au-NPs_25_ group. Similarly, the *Trk-b* gene was up-regulated in both Au-NPs groups, although the upregulation was found to be statistically insignificant (*P*>0.05), with higher expression observed in the Au-NPs_50_ group relative to the Au-NPs_25_ group, as depicted in Table 6.

**Table 6:** Gene expression of *Rgc32* and *Trk-b* in rat after Au-NPs administration

Groups parameters	Control		Au-NP_50_		Au-NP_25_		*P-*value#
	Mean	SD	Mean	SD	Mean	SD	
***Rgc32***	1.000	0.000	3.526	1.93	5.872	5.04	0.050*
***Trk-b***	1.000	0.000	3.898	3.84	2.928	2.61	0.163

Au-NP_50_, gold nanoparticles 50 ppm for 5 days; Au-NP_25__,_ 25 ppm Au-NPs for 10 days; Rgc32, regulator of cell cycle; Trk-b, neurotrophic receptor tyrosine kinase 2. SEM: Standard error of mean, # ANOVA test: *P>0.05.

## Discussion

In the present study, we aimed to investigate the effect of Au-NPs dose versus time in inducing short-term effects on the cerebellum, which, to our knowledge, has not been previously examined. Our findings revealed that the larger dose of Au-NPs caused more structural damage with a shorter duration of exposure compared with the smaller dose with a longer duration. At the behavioral level, we observed decreased mobility in the group that received the larger dose with a shorter duration of exposure, while the group that received the smaller dose with a longer duration showed an increase in supported rearing compared with the control group. Interestingly, the OFT showed signs of anxiety in both groups, albeit with different presentations. No significant locomotor or coordination disability was observed by the NBT or OFT. We also found an increase in the expression of the *Rgc32* gene in both groups compared with the control, with a significant upregulation in the group that received the smaller dose with a longer duration of exposure. The *Trk-b* gene was up-regulated in both groups, indicating a potential role in the cerebellar response to Au-NPs exposure. Overall, our study’s novelty lies in its concurrent investigation of both dose and duration factors, and our findings provide important insights into the potential adverse effects of Au-NPs on the cerebellum.

### Dose-dependent Au-NPs histological alterations in the cerebellum

The gold nanoparticle accumulation in various brain regions (hippocampus, frontal cortex, entire cortex, cerebellum, and hypothalamus) was reported in earlier studies [47]. Inhalation of agglomerates containing 7-nm Au-NPs generated the highest mass concentration of Au-NPs in the lungs, followed by brain regions such as the olfactory bulb, hippocampus, striatum, frontal cortex, entorhinal cortex, septum, cerebellum, aorta, esophagus, and kidney. After inhaling agglomerates of 7-nm, eight organs/tissues, particularly the brain, retained a higher mass concentration of Au [48]. In a previous study, the researchers analyzed the amount of Au-NPs present in the brain tissue at different time points after injection. They found that the overall amount of Au-NPs in the brain tissue was 0.06% of the initial amount injected per gram of brain tissue. However, when they looked at specific time points, they found that the highest amount of Au-NPs per gram of brain tissue was detected at 1-h post-injection (0.29%), and then gradually decreased over time. Despite this, Au-NPs could still be detected in the brain tissue up to 4 weeks after injection. The researchers also conducted a stratified analysis based on follow-up time, which confirmed that the amount of NPs significantly decreased after 72 h compared with the group before 12 h. Overall, this study sheds light on how Au-NPs distribute and clear from the brain tissue over time [23].

The present study revealed that Au-NPs oral administration displayed histopathological distortion with displaced, shrunken, irregular Purkinje cells of the cerebellum in association with decreased purple Nissl’s granules in the perikarya of Purkinje cells. All these changes were associated with a positive caspase 3 reaction in the nucleus of Purkinje cells to different degrees of immunostaining reaction. The noted histopathological changes were dose dependent more than time dependent. In consistence with these findings, Fernanda et al. [24] documented the Au-NPs distribution in mice brain up to 7 days after intravenous injection [24]. The healthy BBB considered as protective physiological mechanisms to protect sensitive neurons from blood-borne nanoparticle exposure [49]. High concentrations of anionic and cationic NPs plays a role in BBB destruction, accompanied by the delivery of NPs to the brain parenchyma, which leads to neurotoxicity [50,51]. Furthermore, the positive proapoptotic marker caspase 3 reaction revealed that Au-NPs with different doses (25, 50, 100, and 250 mg/kg) induced neurotoxicity by generation of oxidative stress and proapoptotic cascade initiation in brain [52]. Other proposed mechanisms for Au-NPs neurotoxicity include epithelial tissue damage [53], inflammation, caspase 3 activation, and an oxidative stress response [52]. These biochemical processes lead to inflammation and DNA damage, which subsequently leads to apoptosis. However, Tiwari and Amiji (2006), claimed that both 50 and 15 nm NPs did not exhibit histological differences in the brain [50]. Similarly, no cytotoxic effect was found in the retina of mice [48] or the brain of rats [54] or brain of rat [55] after exposure to Au-NPs. The explanation for this variation between our research and the findings of others but could be linked to the shape, size, surface chemistry, synthesis methods, surface composition of the NP, duration of exposure and higher concentrations of Au-NPs used [56]. Apoptosis, necrosis, and autophagy mechanisms could be responsible for cytotoxicity and Au-NPs-induced cell proliferation inhibition [57]. Apoptosis could be detected at three levels: genetic, protein, and cytological [58]. Au-NPs’ size is linked to apoptosis levels. Au-NPs with a diameter of approximately 15 nm appeared to induce the most apoptosis. Au-NPs shape influences apoptosis levels as well [57,59,60]. Hexagonal Au-NPs appear to be more likely to induce apoptosis than spherical Au-NPs [57,61,62].

It is to be noted that both intravenous and oral studies have produced conflicting results regarding the toxicity of Au-NPs. This inconsistency can be attributed to several factors, including the species and gender of the tested animals, the administered dose, the mode of delivery, the size, shape, and stability of the NPs used, duration of administration and the organs and parameters tested [63–67].

#### Cerebellum and Behavior

In the current research, we noticed that rats did not show a motor deficit in the form of loss of coordination or inability to walk. Yet, they displayed periods of immobility. Reduced locomotor activity, lessened distance traveled in the central region, diminished percentage of spending time in the central zone, increased percentage of time being spent in the periphery zone, in the corners are all reflecting anxiety-related behavior in the tested animal [68]. Although we did not examine the whole brain or the regions related to anxiety such as medial prefrontal cortex or amygdala [69], one cannot exclude the role of the cerebellum in this issue. The most visible manifestations of cerebellar damage are severe motor deficits such as ataxia and loss of oculomotor control. This has contributed to the popular belief that the cerebellum is primarily involved in motor function, but this is far from a fuller understanding of the cerebellum’s behavioral capabilities. According to functional magnetic resonance imaging (fMRI) studies, some regions of the cerebellar cortex are dedicated to motor function, while others are involved in working memory, language, emotion, executive function, and a variety of other nonmotor functions. Autism spectrum disorder, anxiety, attention deficit disorder, schizophrenia, and other nonmotor neurological disorders have all been linked to the cerebellum [70–72]. Therefore, further studies regarding the effect of Au-NPs on behavior in relation to cerebellum are warranted.

Rearing behavior is a classic exploratory behavior in which the animal stands on its hind legs for a short period [73]. In the present study, we noticed a diminished supported rearing behavior in the smaller dose with larger duration group. This suggests that larger dose with shorter duration could negatively affect the activity of the animals. Supported rearing behavior is thought to be highly correlated with activity. Whilst, the unsupported rearing behavior has been indicated to be anxiety sensitive [73]. Rearing behaviors, which seem to be highly context sensitive and may be used in repeated testing designs, may thus can provide extra measure of anxiety in rodents relevant for behavioral studies [73].

### Significant Rgc32 and insignificant Trk-b upregulation

In the current study, we noticed that the *Rgc32* gene was found to be up-regulated in both groups, with a substantial upregulation in the small dose-longer duration group when compared with the control group. The *Trk-b* gene was found to be up-regulated in both Au-NPs groups with the high dose group showing more up-regulation; yet, this higher expression was found to be insignificant.

Au-NPs were found in the kidney, spleen, liver, intestine, urine, and feces. Smaller NPs had a significant impact on DNA damage in Wister rats [74]. Chronic and non-chronic Au-NPs exposure at small doses both induce gene changes after a prolonged period [75,76]. In an earlier *in vitro* study, surprisingly, non-chronic exposure was found to induce more gene expression changes than chronic exposure, and the stress effects of this type of exposure were sustained even after 20 weeks with no additional NP exposure. The chemistry of NP surfaces was important in changing gene regulation. These findings suggest that cells can adapt to chronic, low-level NP insults; however, the cell stress response is irreversible over time after NP removal in acute, non-chronic exposure [75]. This explanation is consistent with the results of our study. This is because both doses were non-chronic, however, the dose dependent effect was more prominent than the duration-dependent effect in the present study.

## Limitations

The study has limitations due to the small sample size, short sub-chronic duration, and focus on the cerebellum alone, which may impede the identification of additional effects on chronic use and make it unclear whether the observed anxiety during behavioral analysis is specifically related to cerebellar injury or to other brain injuries that affect emotions and behavior. Future research should validate the stability of Au-NPs in physiological solutions in the body.

## Conclusion

The toxicity of Au-NPs is dependent on both the dose and duration of exposure. The present study, to the best of our knowledge, is the first to suggest that a larger dose for a shorter duration may result in more structural damage than a smaller dose administered over a longer period of time. This could be due to a lack of time for cells to adapt when exposed to a large dose in a short period, whereas a smaller dose delivered over a longer period allows for adaptation. Additionally, the impact of cerebellar damage may not be immediately reflected in an animal’s locomotion. Thus, caution is warranted when using Au-NPs in various fields, and the role of the cerebellum in anxiety should not be overlooked when investigating Au-NPs’ cerebellar toxicity. Further research is necessary to fully understand the effects of Au-NPs and their potential impact.

## Data Availability

All data has been displayed in the manuscript. No other data available.

## Abbreviations

Au-NPs: gold nanoparticles
BBB: blood–brain barrier
fMRI: functional magnetic resonance imaging
NBT: narrow beam test
NP: nanoparticle
OFT: open field test
*Rgc32 (Rggc)*: regulator of cell cycle
ROS: reactive oxidative stress
TEM: Transmission Electron Microscopy
*Trk-b (Ntrk2)*: neurotrophic receptor tyrosine kinase 2
UV-Vis: Ultraviolet-Visible spectroscopy analysis
